# The immune escape signature predicts the prognosis and immunotherapy sensitivity for pancreatic ductal adenocarcinoma

**DOI:** 10.3389/fonc.2022.978921

**Published:** 2022-09-06

**Authors:** Hao Lu, Li-Yan Zheng, Ling-Yan Wu, Jun Chen, Na Xu, Sui-Cai Mi

**Affiliations:** Xiamen Hospital of Traditional Chinese Medicine, Xiamen Hospital, Beijing University of Chinese Medicine, Xiamen, China

**Keywords:** Pancreatic ductal adenocarcinoma, immune escape, immune infiltration, prognosis, immunotherapy

## Abstract

**Background:**

Pancreatic ductal adenocarcinoma (PDAC) is one of the deadliest malignancies worldwide. Immune escape is considered to be a reason for immunotherapy failure in PDAC. In this study, we explored the correlation between immune escape-related genes and the prognosis of PDAC patients.

**Methods:**

1163 PDAC patients from four public databases, including The Cancer Genome Atlas (TCGA), International Cancer Genome Consortium (ICGC), Array-express, and Gene Expression Omnibus (GEO), were included in our study. Cox regression analysis was used to identify the 182 immune genes which were significantly associated with overall survival (OS). And then we established an immune escape-related gene prognosis index (IEGPI) score using several datasets as the training cohort and validated it using the validation cohort. Kaplan-Meier (KM) and Cox regression analysis were used to detect the relationship of IEGPI score with OS. We further explored the relationship between the IEGPI and immune indexes. And the prediction value of response for immunotherapy in Tumor Immune Dysfunction and Exclusion (TIDE) dataset.

**Results:**

We establish an IEGPI score based on 27 immune escape genes which were significantly related to the prognosis of OS in PDAC patients. Patients in the high-IEGPI group had a significantly worse overall survival rate compared with that in the low-IEGPI groups by KM curves and cox-regression. 5 of the 32 cancer types in TCGA could be significantly distinguished in survival rates through the low- and high-IEGPI groups. Moreover, the correlation between the IEGPI score was negatively correlated with an immune score in several datasets. And higher IEGPI better recurrence-free survival (RFS) and OS in the patients after patients were treated with both PD-1 and CTLA4 in the public datasets (P<0.05). Intriguingly, by using RT-PCR, we verified that the gene of PTPN2, CEP55, and JAK2 were all higher in the BxPC-3 and PANC-1 than HPDE5 cells. Lastly, we found that the IEGPI score was higher in K-rasLSL.G12D/+, p53LSL.R172H/+, Pdx1Cre (KPC) mice model with anti-PD-L1 than that without anti-PD-L1.

**Conclusion:**

Using the immune escape-related genes, our study established and validated an IEGPI score in PDAC patients from the public dataset. IEGPI score has the potential to serve as a prognostic marker and as a tool for selecting tumor patients suitable for immunotherapy in clinical practice.

## Introduction

Pancreatic ductal adenocarcinoma (PDAC) is a highly malignant exocrine cancer with a dismal prognosis. Although PDAC has a relatively low incidence worldwide, it ranks the fourth leading cause of cancer-related death in the world (1). Radical surgery is the only effective treatment for the cure of PDAC. Unfortunately, most PDAC patients are present in advanced stages when they are diagnosed and not amenable for surgery. Even when PDACs undergo surgery, the 5-year postoperative survival rate is still not ideal. Despite great advances in chemotherapy for pancreatic cancer, chemo-resistance and toxic side effects also inhibited the improvement of PDAC patients’ prognosis after treatment (2). Previous studies have demonstrated the 5-year survival rate of PDAC is less than 9%, which is far lower than that of other malignancies (3, 4).

Recent years, besides chemotherapy (5–7), immunotherapy has become a research hotspot in the treatment of malignances (8, 9), included pancreatic cancer. However, it has a poor effect on pancreatic cancer with PD-1/PD-L1 blockade monotherapy (10). Immune evasion might be the main cause of immunotherapy failure, which reflected in the resistance to immune checkpoint blockade (ICB) therapy (11, 12). More and more studies have disclosed the molecular mechanisms of immune evasion in pancreatic cancer (5, 13, 14), which increases the difficulty in the treatment of PDAC.

Considering the highly lethal characteristic and the poor effect of immunotherapy, PDAC patients’ prognosis should be assessed for surgeons to evaluate the treatment benefits through some immune-related gene markers or signatures before surgery.

In this study, we developed and validated an immune escape-related prognostic signature (IEGPI score) with the whole genome expression data from several datasets for PDAC. More importantly, the IEGPI score could identify PDAC patients with an unfavorable prognostic outcome after surgery, and PDAC patients with a high sensitivity to immunotherapy after received with both PD-1 and CTLA4.

## Materials and methods

### Data source

The gene expression profiles and corresponding clinical data of PDAC were obtained from the TCGA (https://portal.gdc.cancer.gov), ICGC (https://dcc.icgc.org/), Arrayexpress (https://ebi.ac.uk/arrayexpress/), and GEO (GEO, http://www.ncbi.nlm.nih.gov/geo) databases. After we excluded some datasets 1) without overall survival (OS) and survival status; 2) sample size<50, a total of 1137 PDAC patients from seven datasets were enrolled in this study. Six datasets of TCGA-PAAD-US (n=146), ICGC-PACA-AU (n=267), E-MTAB-6134(n=288), GSE71729 (n=125) and GSE57495 (n=63) and GSE62452 (n=66) were used as the training cohorts; one other independent cohort, ICGC-PACA-CA (n=182) was served as an external validation cohort (**Supplementary Table S1**). The Single-cell dataset for PDAC was extracted from CRA001160. Lastly, the immunotherapy cohorts were downloaded from the TIDE (Tumor Immune Dysfunction and Exclusion, http://tide.dfci.harvard.edu/). The expression data of 6 pair KPC mice with PD-L1 and without PD-L1 were downloaded from GSE196435.

### Processing of the immune escape-related genes

Totally, 182 immune escape genes were downloaded from previous study (**Supplementary Table S1**) (15). First, we stratified each immune escape gene into two groups by the median value in 7 datasets. Second, a univariate Cox regression analysis was conducted to filter the 182 immune escape genes which are associated with OS in the PDAC patients. Finally, through the fixed-effects model based on meta-analysis, the pooled hazard ratio (HR) with 95% CI of immune escape gene was estimated.

### Establishing the prognostic immune escape-related genes signature

For those immune escape genes, the pooled HRs with their standard estimates (SE) which were significantly related to prognosis were then integrated as the prognostic immune escape genes weight, and generated the immune escape-related gene prognosis index (IEGPI) score. In sum, IEGPI from a sample is given by:


$$\text{IEGPI score}=\sum_{1}^{i}\left(\frac{HR-1}{SE}\right)*Gene\left(i\right)$$

In the aforementioned formula, gene (i) was the relative expression of OS-related immune escape genes, and n is the total number of OS-related immune escape genes based on the meta-analysis. In our analyses, the normalized Z-score was used to calculate the score.

### Evaluation of tumor immune score

We utilized ESTIMATE (16) to evaluate tumor immune score based on immune gene expression signatures. The immune score, which represents the tumor immune infiltration level, is the fraction of immune cells in bulk tumor.

### Estimation of immune cell infiltration

The single-sample gene set enrichment analysis (ssGSEA) (17) was introduced to quantify the relative infiltration of 28 immune cell types in the tumor microenvironment. Unique feature gene panels for each immune cell subset were obtained from the latest literature (18). An enrichment score in ssGSEA analysis represented the relative abundance of each immune cell type. The ssGSEA score was normalized to unity distribution, for which zero is the minimal and one is the maximal score for each immune cell type. The bio-similarity of the immune cell filtration was estimated by multidimensional scaling (MDS) and a Gaussian fitting model.

### Evaluation of TMB

For each tumor sample, we determined its TMB as the total count of somatic mutations detected in the tumor. TMB was calculated by dividing the nonsynonymous mutations with 38 Mb as previously reported. In addition, we calculated Oncoplot, mutation landscape, and OncogenicPathways based on TCGAmutation and maftools R packages.

### Functional and pathway enrichment analyses

By using the “clusterProfiler” R package (version 4.2.2) (19), the Kyoto Encyclopedia of Genes and Genomes (KEGG) enrichment analysis and Gene Ontology (GO) analysis were performed in our study. Gene Set Enrichment Analysis (GSEA) was used to explore the potential function and signaling pathway enrichment associated with the patients with high and low IEGPI Pancreatic ductal adenocarcinoma cancer.

### RT-qPCR for gene expression study

To gain the insight of the characteristics of IEGIP score, we investigated PTPN2, CEP55 and JAK2 as the represented gene of IEGIP score in the cell lines. The sequences of Primer were set as following rules: PTPN2 Forward Primer was “GAAGAGTTGGATACTCAGCGTC”, and Reverse Primer was set as “TGCAGTTTAACACGACTGTGAT”. CEP55 Forward Primer was “CTTGAGGTTGAACGACAAACCA” and Reverse Primer was “AGCTCTTCGGATCTCTTCTTCTC”. JAK2 Forward Primer was “ATCCACCCAACCATGTCTTCC”, and Reverse Primer was “ATTCCATGCCGATAGGCTCTG”. For cell lines, normal pancreatic ductal epithelial cell (HPDE6-C7 cell, BNCC338285) were purchased from Beijing Beina Chuanglian Institute of Biotechnology (Beijing, China), and PANC-1(GIBCO: 10566016) and BxPC-2(GIBCO: 11875093) were purchased from National Collection of Authenticated Cell Cultures. The condition of RT-qPCR was performed as the following: initial cDNA denaturation was at 95°C for 5 min, and 40 cycles at 95°C for 30s, and annealing temperatures were diversely set at 55°C, and extension at 72°C for 1 min.

### Statistical analysis

Continuous variables were analyzed using the student’s *t*-test or the Wilcoxon rank sum test, while the categorical variables were compared using the χ^2^ or Fisher exact test. The Benjamini–Hochberg (FDR) was also used to adjust the P-values for the multiple comparisons. Immune escape genes with significant prognosis were selected according to the following criteria: meta-analysis, P<0.001, and FDR<0.001. Spearman correlation analysis was used to assess the potential relevance. Univariate and multivariate Cox regression analyses were performed to explore the independent risk factors of OS and recurrence-free survival (RFS) in PDAC patients. Kaplan–Meier (KM) method and log-rank test were performed to compare the OS rates in each group. Time-dependent ROC curve was done to detect the prognostic value of IEGPI for PDAC patients. The area under the receiver operating characteristic curve (AUROC) was used to estimate the diagnostic value of IEGPI for chemosensitivity. All statistical analysis of all the clinical data was performed in R (version 3.6.2; https://www.r-project.org/). A two-sided P-value< 0.05 was considered statistically significant in all tests.

## Results

### Immune escape genes associated with prognosis of PDAC

**Figure 1:**
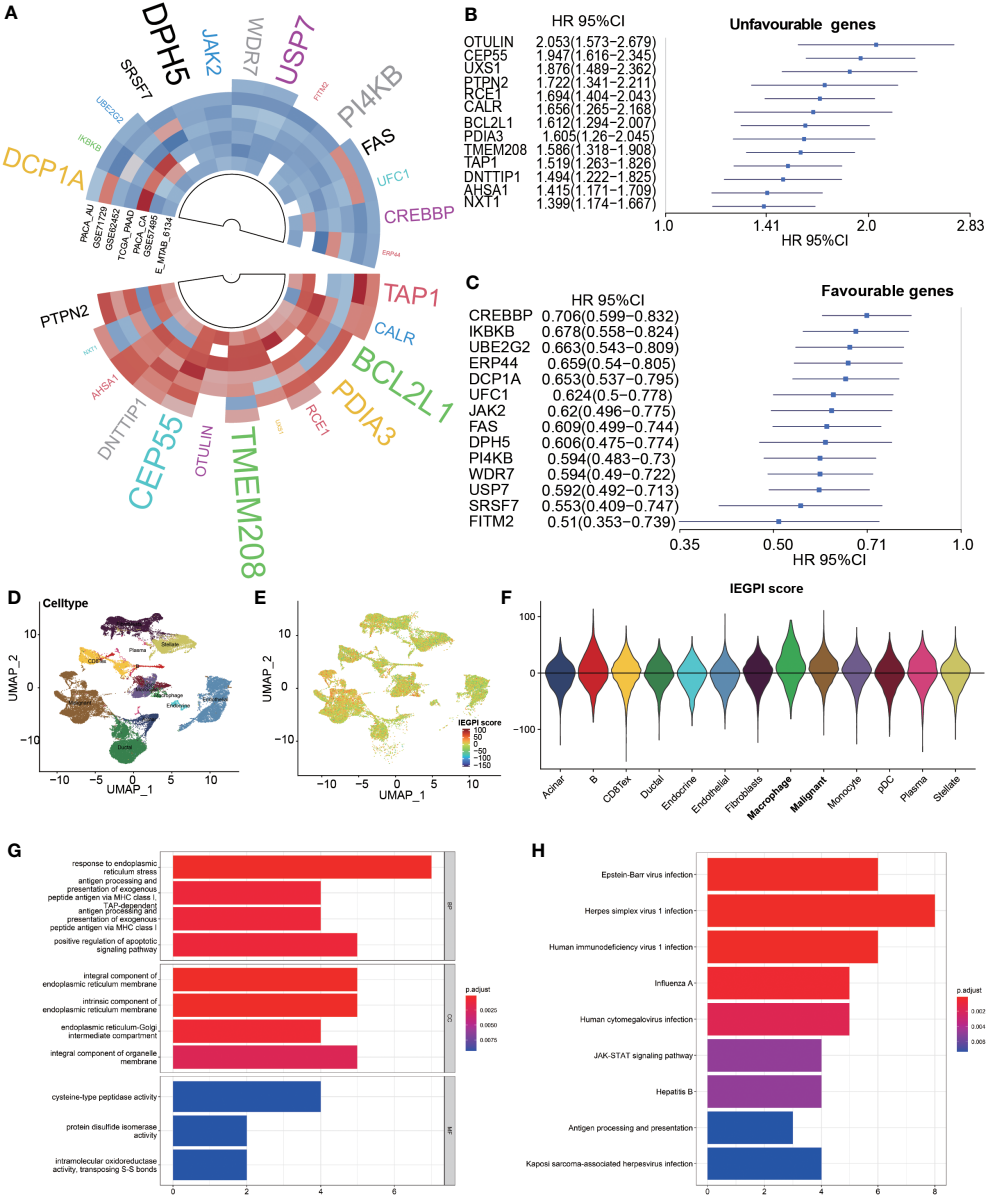
Construction of the IEGPI score. **(A–C)** 27 prognostic immune escape genes; **(D–F)** IEGPI score distribution in single-cell sequencing dataset of CRA001160; **(G)** 27 immune escape genes were implemented into GO analyses; **(H)** 27 immune escape genes were implemented into KEGG analyses.

First, in 7 datasets, we used the Cox regression analyses to uncover the 182 immune escape genes which were significantly associated with OS in PDAC patients. The detail information was list in **Supplementary Table S1**. Second, a meta-analysis (random effects model) was implemented among 7 datasets to harvest pooled HRs and the coefficients of significant genes associated with OS. Finally, we identified 27 immune escape genes which were significantly related to prognosis of OS in PDAC patients (**Figures 1A**). Of them, 13 were poor prognosis-related genes, and 14 were good prognosis-related genes. The forest plots in **Figures 1B, C** showed the pooled HRs and 95%CI of the above 27 immune escape genes after a meta-analysis (**Supplementary Table S2**).

According to the aforementioned formula, we used the 27 immune escape genes to establish an integrated score, namely IEGPI, for every patient in the seven datasets. Their KEGG pathway enrichment results showed in the **Supplementary Table S3**. Using the single-cell sequencing dataset of CRA001160, we found that IEGPI score was elevated in macrophages and tumor cells compared with other groups in a single-cell analysis (**Figures 1D–F** and **Supplementary Table S4**). Additionally, 27 immune escape genes were implemented into GO and KEGG analyses, and the results were illustrated in **Figures 1G, H**. We found some important items were enriched after GO analysis, such as response to endoplasmic reticulum stress, integral component of endoplasmic reticulum membrane, and cysteine-type peptidase activity. Moreover, some significant pathways such as antigen processing and presentation, JAK-STAT signaling pathway were enriched.

### Prognosis estimation of IEGPI score

**Figure 2:**
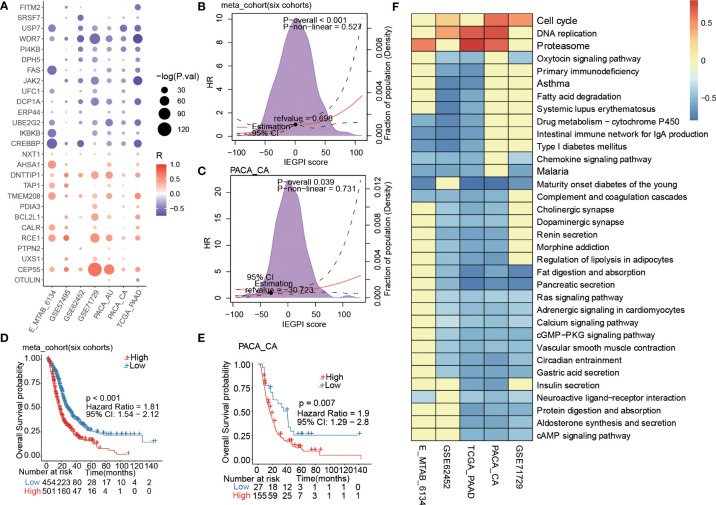
Prognostic estimation of IEGPI score. **(A)** Association of the IEGPI score with the 27 prognostic immune escape genes; **(B, C)** the HR value distribution with different IEGPI score in the training and validation cohorts; **(D, E)** Kaplan-Meier curves of IEGPI score in the training cohort and validation cohorts; **(F)** Relationship between the IEGPI score and cell signaling pathways *via* KEGG analyses.

A correlation plot in **Figure 2A** demonstrated that there existed a strong correlation between the IEGPI score and most of the 27 immune escape-related genes in PDAC patients. **Figures 2B, C** showed relationship between HR value (Cox regression analysis of OS) and IEGPI score in the training cohort and validation cohort, respectively, which reflected a near-linear correlation overall.

More importantly, we merged six datasets of PDAC patients and then used the ROC curve finding the best cut-off value to divide patients into two groups (the high-IEGPI vs. low-IEGPI group). We make a KM curve to show the different OS rates between the high-IEGPI and low-IEGPI group (**Figure 2D**). Patients from the low-IEGPI group had a significantly better survival OS than those from the high-IEGPI group (P<0.001). **Supplementary Figure 1** showed the survival differences between the high-and low-IEGPI groups in six different datasets, which also illustrated the same results with the **Figure 2D**. In addition, a validation cohort of PACA-CA was stratified into two groups used the above cut-off value. The KM curves in PACA-CA cohort also illustrated significantly different OS rates between the two groups (P=0.007) (**Figure 2E**). In addition, we analyzed the relationship between the IEGPI score and cell signaling pathways in five datasets through KEGG analysis (**Figure 2F**).

**Figure 3:**
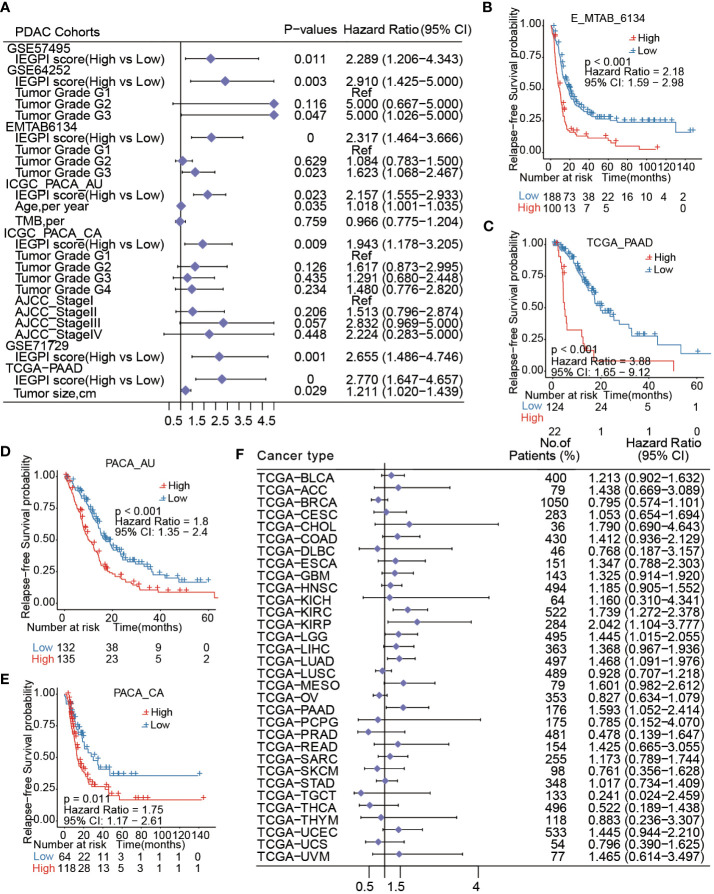
IEGPI score was an independent risk factor of OS in PDAC. **(A)** Multivariate Cox regression analysis results in seven PDAC datasets; **(B–E)** Kaplan-Meier curves of relapse-free survival in PDAC patients with different IEGPI score level in four public datasets; **(F)** the prognostic values of the IEGPI score in the Pan-cancer datasets.

Furtherly, a forest figure in **Figure 3A** showed the multivariate Cox regression analysis results in seven PDAC datasets (**Supplementary Tables S5**, **S6**). We found that IEGPI score was an independent risk factor of OS in all datasets (all P< 0.05). Then we explored the impact of IEGPI score on tumor relapse of PDAC patients in four datasets (E-MTAB-6134, TCGA−PAAD, PACA_AU, and PACA_CA). the results in **Figures 3B–E** demonstrated that patients in the low-IEGPI group had significantly better relapse-free survival rates compared with those in the high-IEGPI groups (P<0.05).

At last, the TCGA pan-cancer analysis found that 5 types of 32 cancers could be significantly distinguished in survival through the low- and high-IEGPI groups (**Figure 3F**), suggesting the application potential of IEGPI score for prognostic prediction in other malignant diseases.

### Relationship between the mutation genes and IEGPI

**Figure 4:**
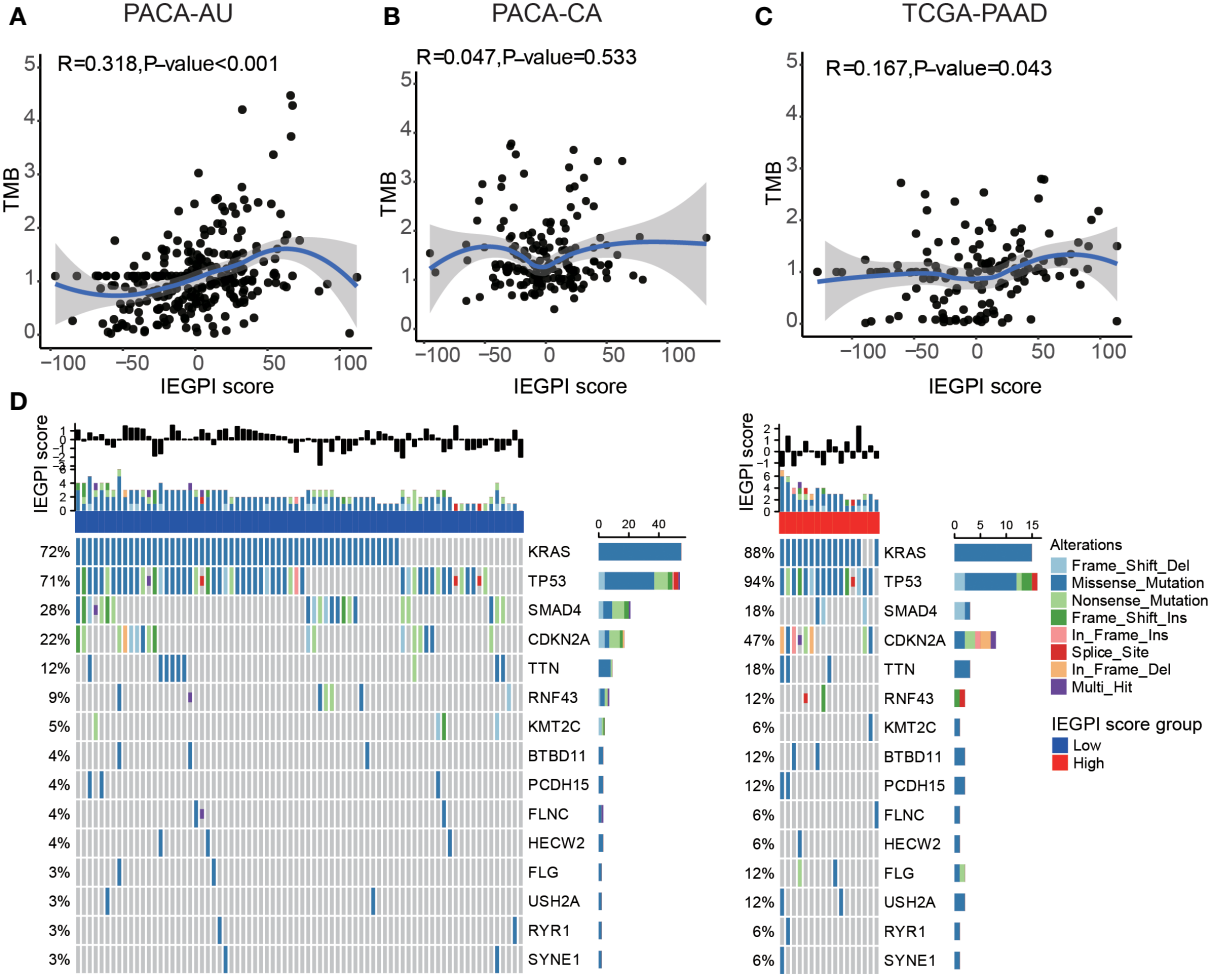
Relationship between the mutation genes and IEGPI score. **(A–C)** Relationship between the IEGPI score and TMB score in three public datasets; **(D)** the landscape of top 15-gene mutations in different IEGPI groups.

We explored the relationship between the IEGPI score and the TMB (tumor mutation burden) value. **Figures 4A–C** showed that in the PACA-AU cohort, PACA-CA cohort, and TCGA-PAAD cohort, the correlation between the IEGPI score TMB was not vary strong.

We then used the Oncoplot to illustrated the top 15 mutation genes in the High-and low-IEGPI score groups of PDAC, and the results included KRAS, TP53, SMAD4 and CDKN2A, etc. (**Figure 4D**). Notably, we observed that TP53 had a significantly higher mutation frequency in High-IEGPI group than in low-IEGPI group (**Figure 4D**). It could explain why High-IEGPI group had the worst prognosis among the two groups since TP53 mutations have been associated with unfavorable outcomes in various cancers.

### Relationship between the immune infiltration and IEGPI

**Figure 5:**
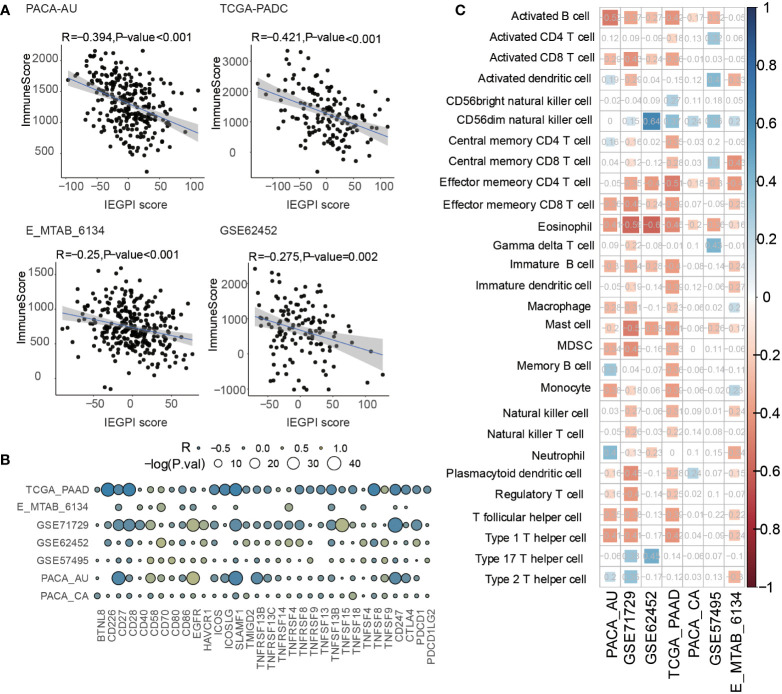
Relationship between the immune infiltration and IEGPI score. **(A)** Relationship between the IEGPI score and immune score in four public datasets; **(B)** correlation of the IEGPI score with immune co-stimulation and check-point genes in seven datasets; **(C)** Relationship between the expressions of 28 immune cells and IEGPI score in seven datasets.

In order to explore the distribution of immune components in different IEGPI score patients, we used the Spearman correlative analysis to explore the relationship between the IEGPI score and the immune score. **Figure 5A** showed that in the PACA-AU cohort, TCGA-PADC cohort, E-MTAB cohort, and GSE62452 cohort, the correlation between the IEGPI score was negative linearly correlated with immune score.

In addition, we selected immune genes based on previous studies to study the relationship of the IEGPI score and immune functions (immune co-stimulation and check-point genes). Bubble plot of **Figure 5B** showed that in seven cohorts, immune genes had a huge-difference relationship with the IEGPI score. Moreover, a total of 28 infiltration immune cell were explored to show their negative relationship with the IEGPI score in in seven cohorts (**Figure 5C**).

### IEGPI score for immunotherapy response prediction

**Figure 6:**
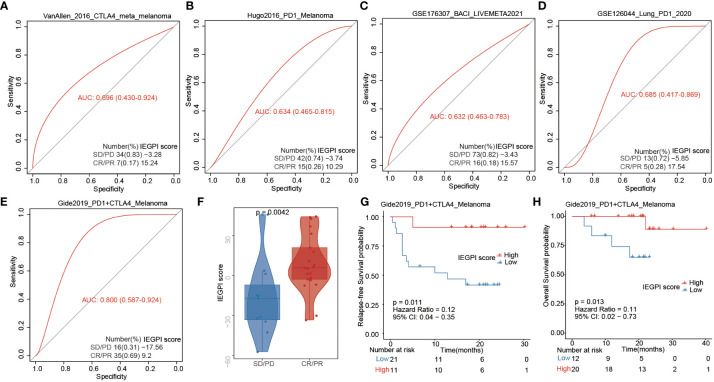
IEGPI score for immunotherapy response prediction. **(A–E)** IEGPI score’s potential as a tool for immunotherapy response prediction in melanoma, bladder cancer, and lung cancer; **(F)** the distribution of IEGPI score in different immunotherapy response groups with both PD-1 and CTLA4 treatment; **(G, H)** Kaplan-Meier curves of patients with different IEGPI score.

First, using the TIDE dataset, we want to evaluate the IEGPI score’s potential as a tool for immunotherapy response prediction in other tumors, such as melanoma, bladder cancer, and lung cancer (**Figures 6A–E**). We found that in the melanoma cohort treated with both PD1 and CTLA4, the likelihood of predicting a positive immunotherapy response is strong with AUC=0.800. Moreover, we also explored the distribution of IEGPI score in CR/PR and PD/SD, and the results showed that the patients with CR/PR had a higher IEGPI score (P<0.05) in melanoma patients receiving the both PD1 and CTLA4 treatment (**Figure 6F**). Additionally, the same trend was found in other tumors, although there were no significant differences between the high and low IEGPI score patients (**Supplementary Figure S2**). Noteworthy, **Figures 6G, H** showed that high-IEGPI score was associated with better RFS and OS in the melanoma patients treated with both PD-1 and CTLA4 (P<0.05).

### Validation of IEGPI *in vitro* and vivo

**Figure 7:**
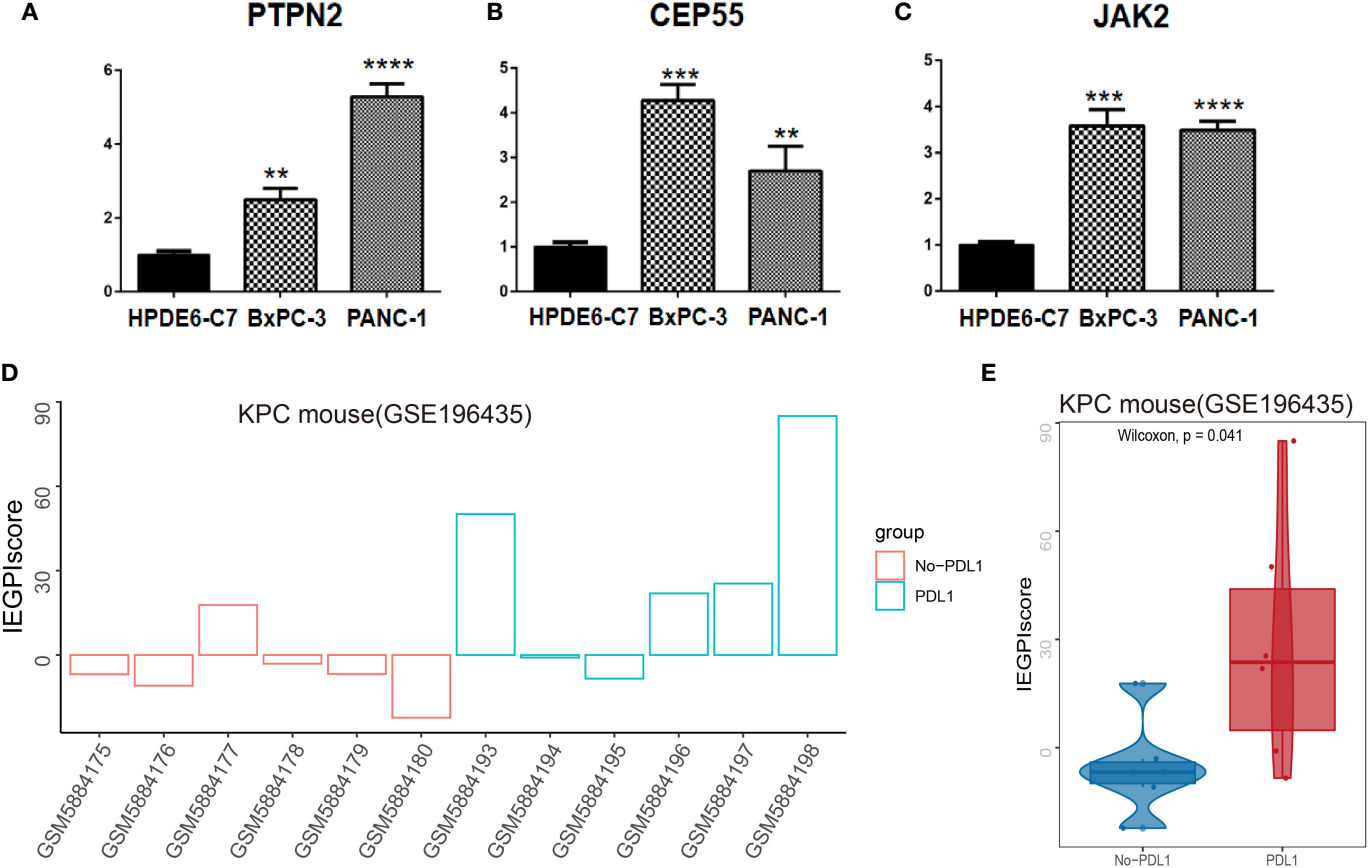
Validation of IEGPI score in cell lines and KPC mouse model. RT-PCR for represented IEGPI genes in HPDE6-C7, BxPC-3 and PANC-1. **(A)** PTPN2, **(B)** CEP55, **(C)** JAK2. **(D)** IEGPI score expression of each sample in the KPC mice model with and without PDL1.**(E)** differences of IEGPI score between the KPC mice model with and without PDL1. **P <0.01, ***P <0.001, ****P <0.0001.

To validate the reality of IEGPI score, we accessed the gene expression of PTPN2, CEP55 and JAK2 as the represented gene of IEGPI score by using RT-qPCR (**Figures 7A–C**). PTPN2, CEP55 and JAK2 were all higher in the PDAC related cell lines than normal cell lines *in vitro*. *In vivo*, we calculated the IEGPI in 12 samples of KPC mouse model and found that IEGPI score was higher in the s KPC mouse with PDL1 than that without PDL1 (**Figure 7D, E**).

## Discussion

PDAC, as the most common type of pancreatic cancer, has an extremely poor survival (20). A mount of efforts has been underway to find an effective solution to improve the long-term prognosis. Blocking the immune checkpoints, such as targets programmed death 1 or programmed death 1 ligand 1 (PD-1/PD-L1) have been considered as a potential therapeutic target against PDAC. However, the treatment effect of pancreatic cancer is not promising with a single PD-1/PD-L1 blockade (21). Immune escape might be one of the reasons that impaired the PD-1/PD-L1 treatment effect of PDAC.

Recently, immune escape has been considered as an important factor which gives rise to immunotherapy failure in cancer therapy. A variety of factors, such as antigenic variation, lacking infiltration of T cells, and changes in tumor microenvironment, are all associated with immune escape (22). Although more and more mechanisms of immune escape have been found in studies for kinds of cancers recently, no study focus on the relationship of immune escape with the prognosis of cancers. In our study, we identified 27 immune escape-related genes which were significantly correlated with the OS in PDAC using the public datasets. More importantly, we selected the training cohort to establish a scoring system, namely IEGPI, which was used to stratify PDAC patients into two groups with significantly different survival outcomes. Then a validation cohort was used to validated and evaluate the prognosis prediction of IEGPI score in PDAC, with a good survival prediction ability in PDAC. Interestingly, the elevated activities of antigen processing and presentation, JAK-STAT signaling pathways have potential contributions to the increased anti-tumor immunity in high-IEGPI score. In addition, we explored the relationship between the TMB, immune infiltration modules and the IEGPI score. Last but not least, we evaluated the ability of IEGPI score for the prediction of immunotherapy response in several cancers, and found that more patients harvested CR/PR have a high IEGPI score, suggesting they achieved a good survival outcome after immunotherapy.

The highlight of this study was that IEGPI score could predict the survival of PDAC patients after surgery, and also could be used as an indicator to reflect the sensibility and response of immunotherapy after receiving the PD-1 and CTLA4 treatments in tumors.

Previous reports have found that inherently immunosuppressive exists in PDAC which leading to a evasion from immune surveillance (23–27). Recently, Wang et al. concluded that the cancer Forkhead box protein 3 (C-FOXP3) can directly activates PD-L1 and mediate the immune escape of PDAC (28). In 2020, Yamamoto et al. identified NBR1-mediated selective macroautophagy/autophagy of MHC-I as a novel mechanism that facilitates immune evasion by PDAC cells (29). In our study, we found 27 immune escape-related genes which were significantly associated with OS in PDAC patients. Immune evasion leads to a poor response to immune checkpoint inhibitors and a poor survival in PDAC. Those genes might be the key genes on which the immunotherapy agents target in the future.

Identified the key immune escape-related genes which are significantly associated with PDAC patient’s prognosis is an important method to assess and improve the effect of immunotherapy. In addition, molecular biomarkers based on immune escape-related genes has improved prognosis estimation for PDAC in daily clinical practice (30, 31).

In recent years, researchers have developed a series of evaluation systems used to assess or identify the progression or prognosis risk of pancreatic cancer using the gene expression datasets. For example, Using WGCNA, Giulietti et al. identified several genes (CEACAM1, MCU, VDAC1, CYCS, C15ORF52, TMEM51, LARP1 and ERLIN2) that appear to be critical to PDAC development, which might be potential therapeutic targets with clinical utility (32). In addition, a study built a prognostic score with 20 genes (PPS20) from high-throughput transcriptomic data in pancreatic cancer and found PPS20 was a more robust transcriptomic signature in prognostic prediction (33). Moreover, a robust 25-gene classifier associated with post-operative OS in pancreatic cancer was identified. It was proved to have a good prognostic value after multivariate analysis (34). However, aforesaid scores or systems don not pay attention to any genes related to immune escape in pancreatic cancer. In our study, we selected immune escape-related genes and developed an IEGPI score to predict the prognosis and distinguish the survival risk of patients.

Collectively, our study firstly integrated 27 prognostic immune escape genes to establish an IEGPI score and validated the expression of PTPN2, CEP55 and JAK2 in cell lines, which shows a good prediction in the prognosis of PDAC and immunotherapy response. The IEGPI might represent a potential prognostic biomarker as well as therapeutic targets of immunotherapy in the future. The higher the IEGPI score is, the higher the level of immune escape is, the worse the killing effect of the body’s immune system on tumor cells is, and the worse the prognosis is. However, when patients received immunotherapy, patients with higher IEGPI score were more sensitive to immunotherapy. Because immunotherapy alters the tumor microenvironment, these individuals have a higher CR/PR ratio and better prognosis. Therefore, our IEGPI score also can be used to screen clinical PADC patients for immunotherapy, allowing them to achieve a better prognosis.

In conclusion, basing the public datasets, our study established and validated an IEGPI score which was an independent risk factor of OS in PDAC, and patients in the high-IEGPI group had a worse survival rate after surgery. Using the TIDE datasets, we also found that in melanoma patients who received the PD-L1 and CTLA4 treatments, high IEGPI-score patients had a better OS and RFS. Above results suggested that our IEGPI score has the potential to serve as a prognostic marker and as a tool for selecting tumor patients suitable for immunotherapy in clinical practice.

## Data availability statement

The original contributions presented in the study are included in the article/**Supplementary Material**. Further inquiries can be directed to the corresponding author.

## Author contributions

HL, L-YZ, L-YW and S-CM performed the research. S-CM, and HL designed the research study. HL, L-YZ and L-YW contributed collection and assembly of data. HL, L-YZ and L-YW analysed the data. HL, L-YZ and L-YW wrote the paper. All authors contributed to the article and approved the submitted version.

## Conflict of interest

The authors declare that the research was conducted in the absence of any commercial or financial relationships that could be construed as a potential conflict of interest.

## Publisher’s note

All claims expressed in this article are solely those of the authors and do not necessarily represent those of their affiliated organizations, or those of the publisher, the editors and the reviewers. Any product that may be evaluated in this article, or claim that may be made by its manufacturer, is not guaranteed or endorsed by the publisher.

## Supplementary material

Supplementary Figure 1 Kaplan-Meier curves of IEGPI score in six datasets involving in the training cohort.

Supplementary Figure 2 The distribution of IEGPI score in different immunotherapy response groups.
