# Supplementary figures and images for: The immune escape signature predicts the prognosis and immunotherapy sensitivity for pancreatic ductal adenocarcinoma
Source: Front Oncol. 2022 Sep 6;12:978921. doi: 10.3389/fonc.2022.978921 (PMC9486201; doi:10.3389/fonc.2022.978921)

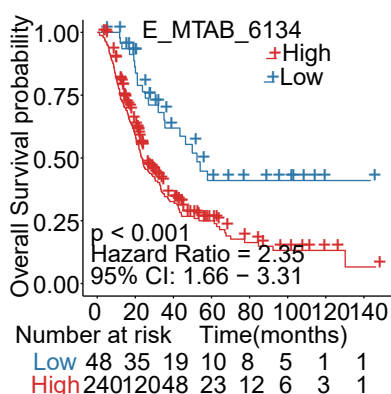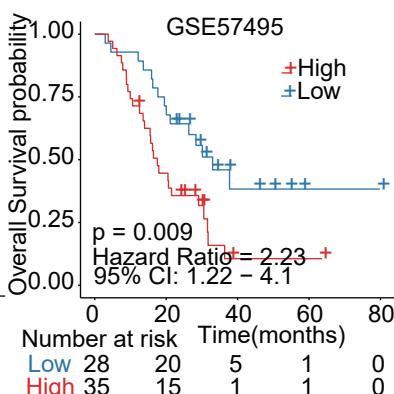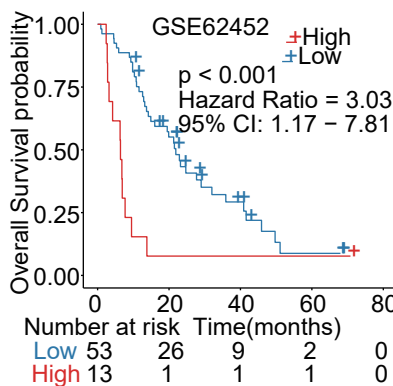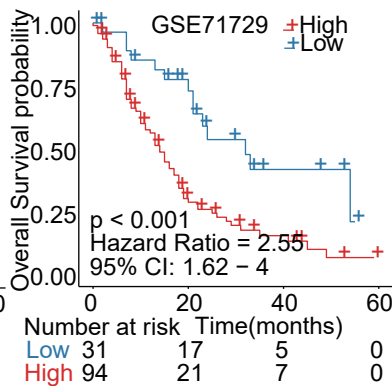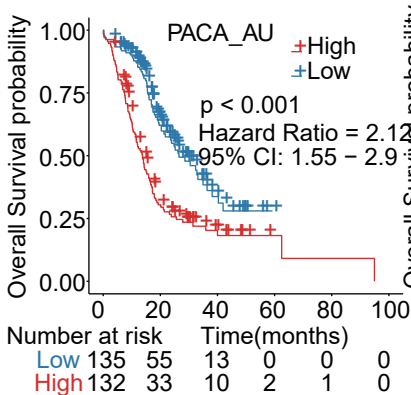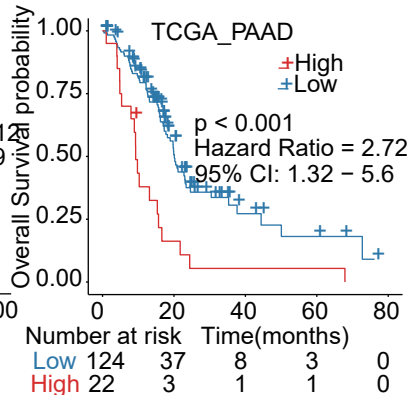

Supplement: Supplementary Figure 1 — Kaplan-Meier curves of IEGPI score in six datasets involving in the training cohort. [file Image_1.pdf]

A

VanAllen(2016)\_CTLA4\_meta melanoma

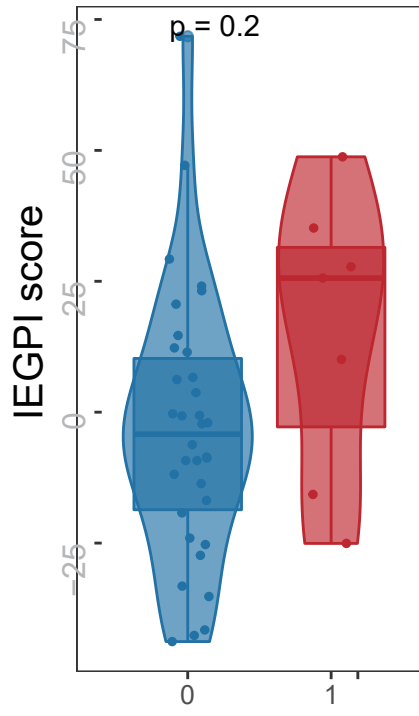

B

Hugo(2016)\_PD1\_Melanoma

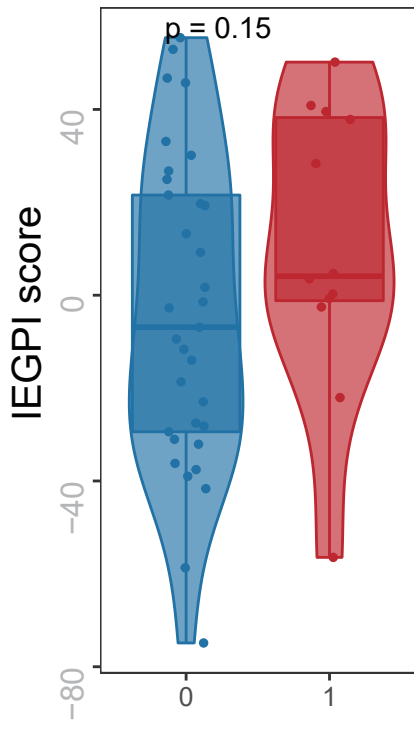

C

GSE176307(2021)\_ICB\_BACI

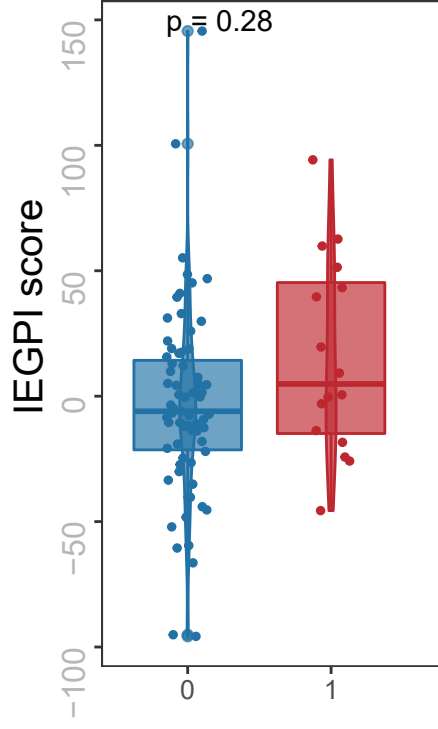

D

GSE126044(2020)\_Lung\_PD1

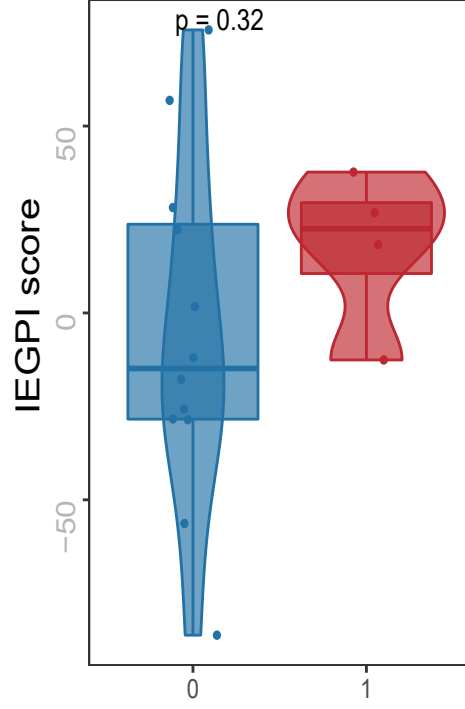

Supplement: Supplementary Figure 2 — The distribution of IEGPI score in different immunotherapy response groups. [file Image_2.pdf]
